# *Histoplasma* antigen detection: A laboratory-based retrospective review of routine testing from 2021 to 2023

**DOI:** 10.7705/biomedica.7866

**Published:** 2026-04-28

**Authors:** Juan Carlos Gómez, Diego H. Cáceres

**Affiliations:** 1 Mycology laboratory, SYNLAB Colombia, Medellín, Colombia SYNLAB Colombia SYNLAB Colombia Medellín Colombia; 2 IMMY, Norman, OK, United States of America IMMY IMMY Norman, OK United States of America; 3 Grupo de Estudios en Microbiología Traslacional y Enfermedades Emergentes - MICROS, Escuela de Medicina y Ciencias de la Salud, Universidad del Rosario, Bogotá, D.C., Colombia Universidad del Rosario Grupo de Estudios en Microbiología Traslacional y Enfermedades Emergentes - MICROS, Escuela de Medicina y Ciencias de la Salud Universidad del Rosario Bogotá, D.C. Colombia; 4 Center of Expertise in Mycology Radboudumc/CWZ, Nijmegen, The Netherlands Center of Expertise in Mycology Radboudumc/CWZ Center of Expertise in Mycology Radboudumc/CWZ Nijmegen The Netherlands

**Keywords:** Histoplasma, histoplasmosis, antigens, diagnosis, Colombia., Histoplasma, histoplasmosis, antígenos, diagnóstico, Colombia.

## Abstract

**Introduction.:**

Progressive disseminated histoplasmosis is associated with high mortality in people with advanced HIV (Human Immunodeficiency Virus) disease or severe immunosuppression. The World Health Organization (WHO) recommends antigen testing for progressive disseminated histoplasmosis diagnosis.

**Objective.:**

To assess the positivity of the *Histoplasma* antigen test in urine specimens referred to SYNLAB.

**Materials and methods.:**

A retrospective analysis of SYNLAB’s database included specimens referred from February 2021 to August 2023. Specimens were tested using the Clarus *Histoplasma* GM Enzyme Immunoassay™.

**Results.:**

A total of 2442 urine samples were tested, of which 282 (12%) were positive, with a higher positivity rate in adults older than 18 years (12%), compared to patients younger than 18 years (4%). The median *Histoplasma* antigen concentration was 9.25 EIA (Enzyme Immuno-Assay) units, significantly higher in adult patients (9.6 EIA units) than in patients younger than 18 years (1.45 EIA units). Testing rates increased over time, peaking in the first quarter of 2023 with 358 tests done, including 25 specimens that tested positive (7%).

**Conclusion.:**

Overall, we found that one in four patients tested positive. Higher positivity and antigen concentration were observed in adults. These findings highlight the importance of antigen testing in diagnosing progressive disseminated histoplasmosis, particularly in high-risk populations.

## Resumen

**Introducción.:**

La histoplasmosis diseminada progresiva está asociada con la mortalidad de personas con HIV (*Human Immunodeficiency Virus*) en etapas avanzadas o en pacientes con inmunosupresión grave. La Organización Mundial de la Salud (OMS) recomienda practicar pruebas de detección del antígeno para el diagnóstico de la histoplasmosis diseminada progresiva.

**Objetivo.:**

Evaluar la positividad de la prueba de detección del antígeno para *Histoplasma* en muestras de orina remitidas al SYNLAB.

**Materiales y métodos.:**

Se realizó un análisis retrospectivo de la base de datos del SYNLAB con las muestras remitidas entre febrero del 2021 y agosto del 2023. Las muestras fueron analizadas utilizando el inmunoensayo enzimático Clarus *Histoplasma* GM™.

**Resultados.:**

Se analizaron 2442 muestras de orina, de las cuales 282 (12 %) fueron positivas, con una mayor tasa de positividad en los pacientes mayores de 18 años (12 %) en comparación con los menores de 18 años (4 %). La mediana de concentración del antígeno para *Histoplasma* fue de 9,25 unidades EIA (*Enzyme Immuno-Assay*), significativamente mayor en pacientes adultos (9,6 unidades EIA) que en los menores de 18 años (1,45 unidades EIA). Las tasas de pruebas aumentaron con el tiempo, alcanzando su punto máximo en el primer trimestre del 2023 con 358 pruebas realizadas, incluidas 25 muestras positivas (7 %).

**Conclusión.:**

En general, se encontró que uno de cada cuatro pacientes analizados resultó positivo. Se observó una mayor positividad y concentración del antígeno en adultos. Estos hallazgos destacan la importancia de las pruebas para detectar el antígeno en el diagnóstico de la histoplasmosis diseminada progresiva, particularmente en poblaciones de alto riesgo.

Histoplasmosis, caused by the thermal dimorphic fungus *Histoplasma capsulatum*, is a significant infectious disease with global implications ^1^^-^^4^. While cases have been reported worldwide, the disease is endemic in specific regions, notably in the Americas, Africa, and parts of Asia ^1^^-^^4^. In recent decades, the relevance of histoplasmosis has grown exponentially, largely due to the increasing number of cases associated with immunocompromised individuals ^5^^-^^8^. Among these, people with advanced HIV (Human Immunodeficiency Virus) disease are particularly vulnerable, though cases have also been documented in organ transplant recipients and other patients with severe immunosuppression ^1^^-^^8^.

In Colombia, histoplasmosis is considered a major endemic mycosis with a wide geographic distribution. Surveillance studies and retrospective analyses have identified histoplasmosis as one of the leading causes of opportunistic infections among people with HIV/AIDS, often presented as a disseminated and life-threatening condition. Despite its high clinical impact, the disease remains underdiagnosed and frequently misclassified due to nonspecific clinical features and limited access to rapid diagnostic methods ^2^^,^^9^^-^^12^.

The timely and accurate diagnosis of histoplasmosis, particularly its progressive disseminated form, is critical for effective treatment and improved patient outcomes ^3^^,^^6^^,^^13^^-^^19^. Traditional diagnostic methods, such as culture and histopathology, while valuable, have notable limitations ^20^^-^^22^. These include prolonged turnaround times and variable sensitivity, which can delay appropriate medical intervention ^6^^,^^23^^-^^25^. As a result, there has been a significant push towards the development and adoption of new rapid diagnostic tests.

In recent years, commercial kits for the detection of *Histoplasma* antigen have become widely available ^26^^-^^33^. The medical community has quickly embraced antigen detection methods as key tools for diagnosing histoplasmosis. Antigen testing offers rapid results with high accuracy, facilitating early and targeted therapeutic interventions that are crucial for patients with compromised immune systems.

The World Health Organization (WHO) has recognized the diagnostic challenges posed by histoplasmosis and the potential benefits of *Histoplasma* antigen testing ^1^^,^^34^. In 2022, the WHO endorsed the use of assays based on *Histoplasma* antigen detection as the first-line diagnostic option for progressive disseminated histoplasmosis in people with advanced HIV disease ^35^^,^^36^. This endorsement underscores a global shift towards integrating antigen testing into routine clinical practice. Furthermore, cost analyses have demonstrated that *Histoplasma* antigen screening is not only effective but also cost-efficient ^7^. Such screening has the potential to prevent up to 17% of AIDS-related deaths in individuals with advanced HIV disease, making it a vital component of public health strategies in endemic regions.

The impact of *Histoplasma* antigen testing extends beyond individual patient care. The implementation of these tests has led to a marked increase in the number of detected cases in various regions around the world ^3^^,^^10^^,^^37^^-^^43^. Additionally, the widespread adoption of these assays has been associated with a significant reduction in mortality rates, particularly in resource-limited settings where access to advanced healthcare is often constrained ^3^^,^^11^^,^^37^^-^^41^^,^^44^^-^^67^.

The ongoing research and advancements in histoplasmosis diagnostics, especially through antigen detection, highlight the critical role of innovation in combating infectious diseases. As global health challenges evolve, the medical community must continue to prioritize the development and implementation of effective diagnostic tools that can improve patient outcomes and reduce mortality rates. Histoplasmosis, once considered a neglected disease, is now receiving the attention it deserves, thanks in large part to these advancements in diagnostic technology ^1^.

Given these developments, the objective of this study was to analyze the positivity rate of *Histoplasma* antigen tests in specimens referred to SYNLAB.

## Materials and methods

A retrospective analysis of the SYNLAB laboratory database was conducted, including all urine specimens submitted for *Histoplasma* antigen testing from February 2021 to August 2023. The specimens were tested using the Clarus *Histoplasma* GM™ enzyme immunoassay (IMMY, reference HGM201), which employs the calibrator cut-off procedure for antigen detection. Testing, analysis, and interpretation were performed in accordance with the kit’s standard operating procedure.

Briefly, the calibrator cut-off procedure uses a user-prepared calibrator, along with a positive control, a negative control, and a blank. The assay measures optical density values, which were used to calculate the antigen concentration. The concentrations of *Histoplasma* antigen in the specimens were reported in enzyme immunoassay (EIA) units. The calibrator cut-off procedure ensures accuracy and reliability in determining antigen levels, supporting accurate diagnosis and monitoring of histoplasmosis cases.

### Statistical analysis

Descriptive statistics were used to summarize demographic and laboratory data. Categorical variables were compared using the *X* ^2^ test or Fisher’s exact test, as appropriate. Continuous variables were presented as medians with IQR (interquartile ranges) and compared using the Mann-Whitney U test. A p < 0.05 was considered statistically significant. Statistical analyses were performed using R software (R Foundation for Statistical Computing, Vienna, Austria).

This project does not involve direct contact with individuals, interventions, or the use of identifiable personal information. As a result, it does not meet the criteria for human subject research. Therefore, it was not required to undergo review by the Institutional Review Board. Since the study solely relies on de-identified data from laboratory records, it falls beyond the ethical oversight typically required for research involving human participants.

## Results

During the analysis period, 2442 urine specimens were tested for *Histoplasma* antigen detection. Of these, the majority-1737 (71%) specimens-were from male patients. The demographic data showed a median patient age of 38 years, with an IQR of 24 years, reflecting a wide age distribution among the tested individuals. Notably, 204 (8%) patients were under 18 years of age, highlighting the importance of testing for histoplasmosis in both pediatric and adult populations. This age range emphasizes the disease’s relevance across various life stages and the need for inclusive diagnostic practices. The study also demonstrated efficient laboratory performance, with a mean turnaround time for results of 3 days, and an IQR of 2 days.

**Figure 1.**
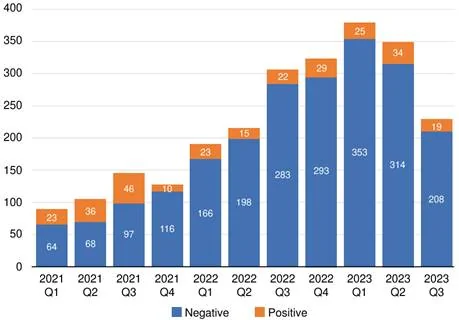
Distribution of antigen test positivity in urine specimens referred to SYNLAB: analysis by quarters, 2021-2023*

An increasing trend in the number of tests done per quarter was observed, with 95 specimens tested in the first quarter of 2021, rising to 213 specimens by the third quarter of 2023. The highest volume of testing occurred in the first quarter of 2023, with 372 specimens analyzed (figure 1). This upward trend indicates a growing demand for *Histoplasma* antigen testing, potentially reflecting increased awareness or incidence of infection during this period.

* The figure shows the number of urine specimens referred and tested for *Histoplasma* antigen each quarter. An increasing trend was observed over the study period, reflecting a rise in test uptake over time.

**Figure 2.**
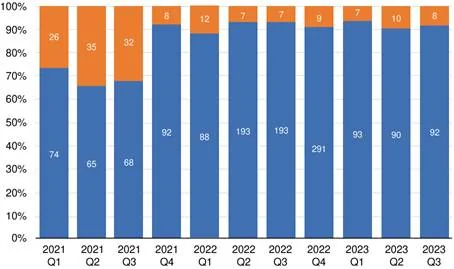
Proportion of positive tests by quarter of samples sent to SYNLAB, 2021-2023*

Out of the 2442 urine specimens tested, 282 (12%) were positive for *Histoplasma* antigen. Among the positive results, 274 (12% of 2238 specimens) from patients older than 18 years, while 8 (4% of 204 specimens) from patients under 18 years (p < 0.001). The percentage of positive tests varied by quarter, ranging from 7 to 35%. Notably, positivity rates were higher in the initial quarters following the implementation of the test. Additionally, the number of specimens tested was lower in these early quarters compared to subsequent periods, which may have influenced the observed positivity rates (figure 2).

* The figure presents the proportion of positive test results per quarter. Higher positivity rates were seen in the early quarters after implementation, potentially influenced by lower specimen volumes and targeted testing in high-risk populations.

**Figure 3.**
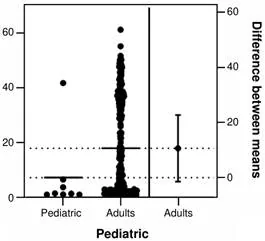
Comparison of *Histoplasma* antigen concentration between pediatric and adult patients*

The median concentration of *Histoplasma* antigen in positive patients was 9.25 EIA units. When stratified by age, the concentration of the *Histoplasma* antigen was significantly higher in patients older than 18 years, with a median of 9.6 EIA units, compared to patients younger than 18 years, who had a median concentration of 1.45 EIA units. This indicates a notable difference in antigen levels between age groups (p = < 0.001) (figure 3).

* This figure illustrates the distribution of antigen concentrations in urine specimens from patients younger than 18 years and those 18 years or older. Median antigen levels were significantly higher in adult patients, suggesting potential differences in disease burden or immune response.

### Ethical responsibilities

This study was conducted in accordance with ethical standards and applicable regulations.

## Discussion

The retrospective analysis specimens referred for *Histoplasma* antigen detection revealed several key insights into the epidemiology and diagnostic patterns of histoplasmosis in a private laboratory. The demographic data indicate a predominantly adult male cohort; a broad age range also highlights the relevance of histoplasmosis across diverse age groups. Notably, 8% of the patients were under 18 years old, underscoring the necessity for inclusive diagnostic practices that address both pediatric and adult populations.

These findings are in line with previously reported data from multiple Latin American countries, where antigen detection has proven instrumental in improving early diagnosis, particularly in people with HIV. For example, Medina *et al*. ^3^ and Cáceres *et al.*^18^ reported that the implementation of rapid diagnostic tests, including *Histoplasma* antigen detection, significantly improved case detection and reduced mortality in Guatemala and across Central America ^3^^,^^18^.

In Colombia, a national survey by Arango *et al*. highlighted underdiagnosis of histoplasmosis, emphasizing the need for better diagnostic tools ^9^. This gap has been partly addressed by interventions such as those described by Cáceres *et al*., where training and implementation of antigen testing led to a marked increase in case identification ^10^.

Similar diagnostic gains were documented in Brazil and Paraguay. Falci *et al*. ^19^ demonstrated that *Histoplasma* antigen detection significantly increased the diagnostic yield compared to conventional methods. In Paraguay, Aguilar *et al*. ^14^ implemented a diagnostic package that included antigen detection, demonstrating its operational feasibility and clinical impact. Likewise, Andreani *et al.*^15^ confirmed the effectiveness of integrated screening for HIV patients in Argentina, where rapid CD4 testing and fungal antigen detection improved early diagnosis ^14^^,^^15^^,^^19^.

Our findings echo these regional experiences, confirming that antigen detection fills a critical gap in clinical and laboratory practice. The test’s performance, especially in non-culture-based settings, allows for more rapid and accurate identification of disseminated histoplasmosis, a disease often overlooked or misdiagnosed. This reinforces the call for broader implementation of antigen-based testing as a standard of care, particularly in private and decentralized laboratories where histoplasmosis remains underrecognized ^7^^,^^16^^,^^43^^,^^68^.

The study’s findings also reflect efficient laboratory performance, with a mean turnaround time of 3 days, indicating timely and reliable diagnostic results. This rapid processing is crucial for effective patient management and early intervention in suspected cases of histoplasmosis. In contrast, the specialized mycology laboratory in Medellín reported significantly longer turnaround times across diagnostic assays, including culture-based methods, and histopathology results ranged broadly, often between 7 and 14 days, with some culture results extending even longer due to slow fungal growth ^23^.

An increasing trend in the number of tests conducted per quarter suggests a growing demand for *Histoplasma* antigen testing, indicating an increased awareness of histoplasmosis, possibly driven by improved diagnostic capabilities or heightened clinical suspicion.

We observed differences in *Histoplasma* antigen concentrations between patients older and younger than 18 years, suggesting that older individuals may experience more severe disease. However, to our knowledge, no published studies have specifically compared antigen levels across age groups, and data on pediatric populations remain particularly limited. This lack of comparative evidence constrains our ability to contextualize these findings within broader epidemiological or clinical frameworks. The absence of such data underscores the need for further research exploring age-related differences in disease severity, antigen burden, and clinical outcomes among both adult and pediatric populations affected by histoplasmosis. Unfortunately, due to the absence of clinical information in our dataset, we were unable to identify specific risk factors or determine the clinical forms of histoplasmosis associated with the observed antigen levels.

Additional studies are warranted to confirm these findings and to elucidate the underlying factors contributing to variations in antigen concentrations across age groups.

Overall, these results emphasize the importance of continued monitoring and diagnostic testing for histoplasmosis across different age groups, as well as the need for sustained public health awareness and clinical vigilance to effectively manage and control this opportunistic infection.

The implementation of *Histoplasma* antigen detection testing represents a key improvement over conventional methods such as culture and microscopy. Traditional diagnostics are often limited by low sensitivity, especially in non-invasive specimens, and by long turnaround times, which can delay treatment. In contrast, antigen testing is rapid, non-invasive, and highly sensitive, making it particularly useful in patients with disseminated disease or severe immunosuppression ^20^^,^^21^. Its integration into routine practice can facilitate earlier diagnosis and improve clinical outcomes. Given these advantages, antigen detection should be prioritized in diagnostic algorithms for histoplasmosis, particularly in endemic regions with limited access to advanced laboratory infrastructure.

We observed an increasing trend in the number of antigen tests requested. We observed that one out of four patients tested got a positive result. In addition, stratified by age, older and younger 18 years, in patients older than 18 years the positivity of the antigen test, and the concentration of the antigen was higher, compared with patients younger than 18 years.
